# Non-functional pancreatic neuroendocrine tumours: emerging trends in incidence and mortality

**DOI:** 10.1186/s12885-019-5543-2

**Published:** 2019-04-08

**Authors:** Junjun Wu, Chi Sun, Enliang Li, Jiakun Wang, Xianping He, Rongfa Yuan, Chenghao Yi, Wenjun Liao, Linquan Wu

**Affiliations:** 1 Department of General Surgery, Second Affiliated Hospital of Nanchang University, No.1 Minde Load, Nanchang, 330006 China; 2 Department of Nursing, Second Affiliated Hospital of Nanchang University, Nanchang, China; 3 Department of General Surgery, Jiangxi Province Pediatric Hospital, Nanchang, China; 4 Department of Breast Surgery, Second Affiliated Hospital of Nanchang University, Nanchang, China

**Keywords:** Non-functional pancreatic neuroendocrine tumours, Epidemiology, Incidence-based mortality, APC, SEER.

## Abstract

**Background:**

Our aim was to determine the epidemiology and recent changes in the trends of non-functional pancreatic neuroendocrine tumours (NF-pNETs) at the population level. In addition, we explored the risk factors that are associated with survival duration.

**Methods:**

Cases were identified form the Surveillance, Epidemiology, and End Results (SEER) Programme database from 2000 to 2014. Data on incidence and incidence-based (IB) mortality for NF-pNET were obtained from this database. Secular trends in age-adjusted incidence and IB mortality were determined by using the Joinpoint Regression program. Data analyses were performed using chi-square tests, Kaplan-Meier curves and Cox proportional hazards regression.

**Results:**

Overall, 4766 patients diagnosed with NF-pNET with a median age of 59 years were identified through our descriptive criteria. Caucasian patients accounted for the majority of the study population, and the proportion of patients with distant disease significantly decreased during our study period. Overall, there was an increase in incidence and IB mortality for NF-pNET; however, the rate of increase decreased during the recent years. In addition, the incidence trends of NF-pNET located in the pancreatic head significantly increased, and rates fo increase in IB mortality for NF-pNET in the pancreatic tail decreased in recent years. Additionally, the 1-, 5-, and 10-year survival rates were 79.0, 51.8, 38.1%, respectively. Furthermore, patient age, tumour grade, stage at diagnosis, tumour size, tumour site and resection were associated with mortality.

**Conclusion:**

Despite increases in incidence and IB mortality, the rate of change in IB mortality for NF-pNET has decreased in recent years. Survival duration displayed a secular increase during the overall period, and the prognosis and survival duration of patients were closely related to the time of diagnosis, age of the patients and size and location of the tumour. Appropriate treatment adjustments based on tumour stage may thus facilitate improvements in patient outcomes.

**Electronic supplementary material:**

The online version of this article (10.1186/s12885-019-5543-2) contains supplementary material, which is available to authorized users.

## Background

In this work, we evaluated the epidemiology and the change of tendency in recent years of non-functional pancreatic neuroendocrine carcinoma at a population level. And use the APC (annual percentage changes) to quantize this change as well as set forth its significance. With advancements diagnosis and treatment, such as improvements in imaging technology, surgical techniques, and adjuvant therapies, overall survival and trends in incidence and mortality for NF-pNETs may change. Our study used annual percent change (APC) to evaluate the incidence and incidence-based (IB) mortality trends as well as the prognostic factors in recent years (2000–2014) for NF-pNETs using a population identified through the SEER Programme database in the United States (US).

Pancreatic neuroendocrine tumour (pNET) is a heterogeneous tumour derived from peptide neurons and neuroendocrine cells [1] that is increasing in incidence year by year. These tumours are more indolent than pancreatic adenocarcinoma [1] and are considered to confer better survival outcomes than pancreatic adenocarcinoma. However, patients with metastatic disease are known to have a poor prognosis [2]. pNETs demonstrate a certain degree of malignancy [3] and have an important status in pancreatic carcinoma.

pNETs can be divided into functional (F-pNET) or non-functional (NF-pNET) pNETs depending on their ability to secrete biologically active hormones and to cause characteristic symptoms [4]. NF-pNETs represent the following three types: NF-pNETs that not produce hormones; NF-PNETs that produce hormones at a low enough level to not cause symptoms; and NF-PNETs that produce hormones such as pancreatic polypeptide, chromogranin A, ghrelin, calcitonin or neurotensin that do not cause symptoms [4].

NF-pNETs are typically indolent and accidentally discovered in most patients [5]. There are no good diagnostic methods to identify this potentially malignant cancer in early stages [3], and this disease is usually diagnosed by pathologic examination after operation or metastasis of the disease itself [6]. Chromogranin A and pancreatic polypeptide (PP) [3, 7] can be useful for diagnosis; nevertheless, NF-pNETs are typically diagnosed at advanced stages because of their indolent nature and slow growth, which cause a delay in symptoms [8, 9].

Regardless of metastatic disease, surgical resection of NF-pNETs is considered beneficial for overall survival [8, 10–12]. The predictors of survival include tumour grade, disease stage, tumour size, histopathological type, and lymph node status [10, 11, 13]. In particular, tumour grade is an important predictor of survival [14, 15].

NF-pNETs account for approximately 2% of all pancreatic malignancies [11]. In addition, there are very few studies, especially population-based studies, on the epidemiology and natural history of NF-pNETs. This might be because of the rarity of the disease and the complexity in its classification, as well as the lack of understanding of clinical and prognostic features of NF-pNETs.

Although there are some studies based on the Surveillance, Epidemiology, and End Results (SEER) Programme using the International Classification of Disease for Oncology (ICD-O)-3 codes (8013/3, 8246/3, and 8150/3), they had some deficiencies. Such as the study by AJ Moser [16], who found that enucleation is equivalent to resection and identified enucleation as a factor contributing to survival benefit. However, with advancements diagnosis and treatment, such as improvements in imaging technology, surgical techniques, and adjuvant therapies, overall survival and trends in incidence and mortality for NF-pNETs may change. Our study used annual percent change (APC) to evaluate the incidence and incidence-based (IB) mortality trends as well as the prognostic factors in recent years (2000–2014) for NF-pNETs using a population identified through the SEER Programme database in the United States (US).

## Methods

### Data source

SEER is an authoritative source of information on cancer incidence and survival in the United States. SEER currently collects and publishes cancer incidence and survival data from population-based cancer registries covering approximately 28% of the U.S. population and is maintained by the National Cancer Institute. The SEER Program is the only comprehensive source of population-based information in the United States that includes stage of cancer at the time of diagnosis, incidence and survival data. The mortality data reported by SEER are provided by the National Center for Health Statistics. The geographic areas of the data was based on the reporting system and population difference. The population data used in calculating cancer rates has through appropriate criteria before abstrcation.

We used a retrospective cohort study to the data from the SEER database, which is in term of the November 2016 submission. Data is obtained from 2000 to 2014 from the SEER 18 registries (San Francisco-Oakland SMSA, Connecticut, Detroit, Hawaii, Iowa, New Mexico, Seattle, Utah, Atlanta, San Jose-Monterey, Los Angeles, Alaska Natives, Rural Georgia, California excluding SF/SJM/LA, Kentucky, Louisiana, Greater Georgia and New Jersey with adjustment for the areas impacted by Hurricanes Katrina and Rita).

The SEER Programme registries routinely collect data on patient demographics, cancer features and cancer-associated treatment. The SEER database has coded surgery intervention as a variable, which mentions whether no surgery or resection is performed. However, details of chemotherapy are not included in this database.

### Study population

We used the SEER database for descriptive analysis and determination of incidence trends. For descriptive analysis, in the SEER database, we used ICD-O-3 codes (8013/3, 8150/3, and 8240/3–8249/3) and site codes (C25.0-C25.4 and C25.7-C25.9) from 2000 to 2014. Patients who were diagnosed within 1 month before death (diagnosis reported on the death certificate or diagnosed at autopsy) were included in the analysis of incidence trends. However, these patients were excluded from survival analyses because survival time in the SEER database is calculated in months and not days, and if we used these data, these cases would be treated to have a survival duration of zero. In addition, we excluded patients who were diagnosed with other primary malignant tumours because we wanted to minimize the chances of cases misdiagnosed as NF-pNET because of metastatic disease to the pancreas. Furthermore, we excluded patients who died due to causes other than NF-pNETs because unrelated causes of death were precluded. The SEER historic stages were used for disease stage classification in our study instead of the American Joint Committee on Cancer staging system, as we did not have access to the latter during our study period. The SEER stage classification provides consistent definitions over time and are as follows: localized (the tumour is confined to the primary site); regional (the tumour has spread to regional lymph nodes); and distant (the cancer has metastasized).

### Statistical analysis

We used SEER*Stat software (version 8.3.4) to analyse our data, including incidence, IB mortality, and survival. All rates were age adjusted according to the 2000 US standard population. Standard mortality statistics were not available because the death certificate does not include the histology of cancer. We obtained IB mortality data by associating the features of the incident cancer to the information on the death certificate. IB mortality enables the stratification of mortality by variables associated with cancer onset.

IB mortality were used. Because in a selected year, NF-pNETs IB mortality is a proportion of the total number of deaths caused by NF-pNETs. These people, defined as those who died of NF-pNETs, must be those who had earlier been diagnosed with NF-pNETs, rather than being diagnosed with NF-pNETs simply because of an autopsy.

We used the Joinpoint Regression Analysis program (version 4.5.0.1) from NCI (https://surveillance.cancer.gov/joinpoint/) to examine trends in incidence and IB mortality for NF-pNET. This program can calculate incidence and IB mortality using a model that is a segmented in a log-linear form. The program can calculate APC in age-adjusted incidence and IB mortality in each segment and present 95% confidence interval (CI) values. The software can represent the slope of the curve in the calculated model, and a linear regression model can be fitted to the last line segment when the incidence is predicted to be stabilised.

For survival analysis, we divided the population into three subgroups by the year of diagnosis as follows: 2000–2004, 2005–2009, and 2010–2014. Median survival and survival rates were calculated for the whole group and the four subgroups. The *P* value reported for the analysis of survival trends is for all the three subgroups. Additionally, we performed survival analysis based on treatment and stage at diagnosis. We used the Kaplan-Meier method to calculate the cumulative survival rates and the log-rank test to compare the survival curves.

Independent predictors of mortality were determined by Cox proportional hazard regression. The covariates analysed included patient age, sex, and ethnicity, tumour grade, stage, size and site and treatment. All *P* values were two tailed, and *P* < 0.05 was considered statistically significant for all tests. Stata (version 11.0; StataCorp LP) was used for all survival and other analyses.

## Results

### General description of patients and Tumours

**Fig. 1:**
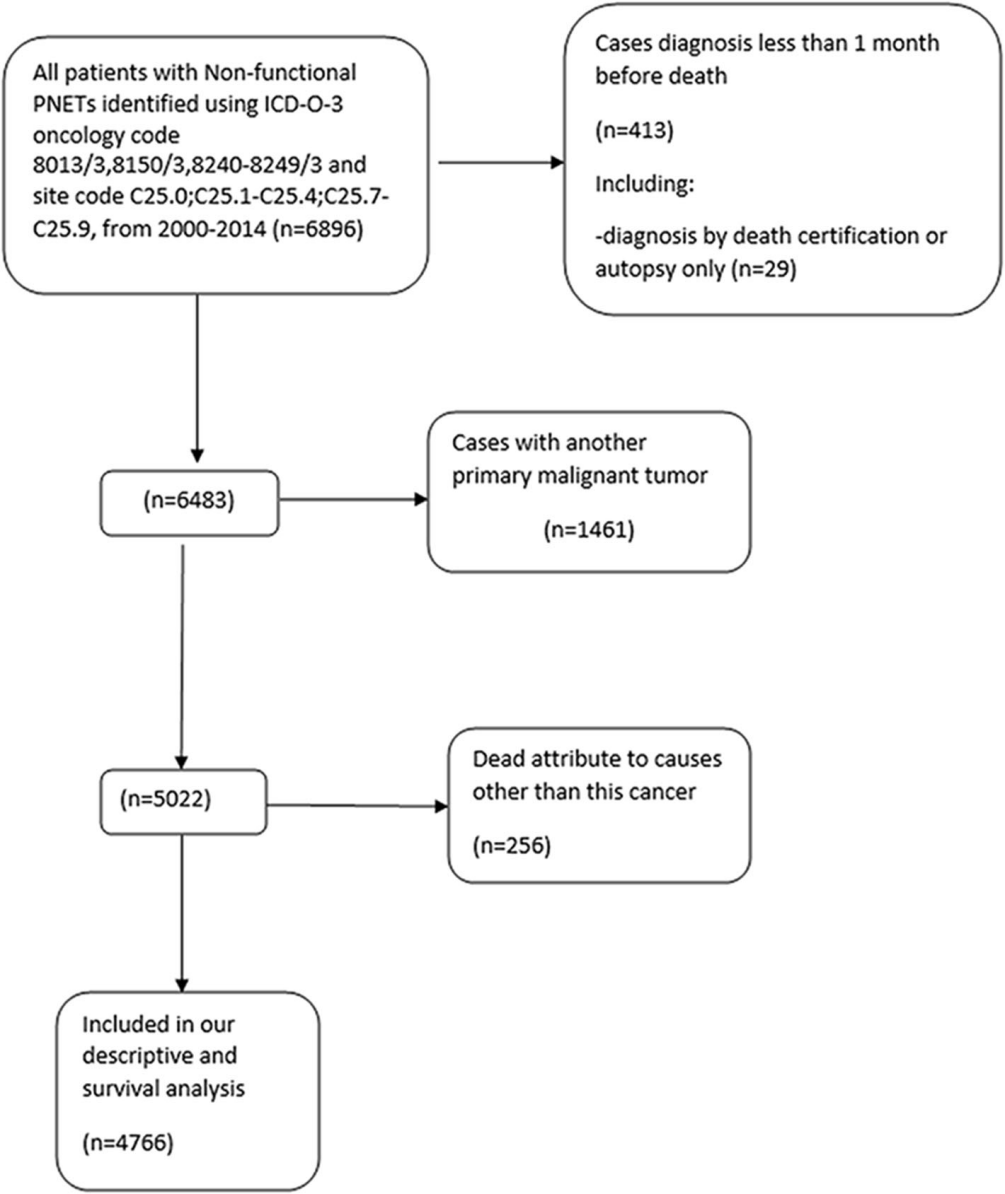
Flow diagram of inclusion and exclusion criteria in our descriptive (excluding incidence trends) and survival analysis within SEER database 2000–2014

We included 4766 patients with NF-pNET using our inclusion criteria for our descriptive and survival analyses (Fig. 1). The demographic and pathological features of the study population are shown in Table 1. The median age at diagnosis in the total cohort was 59 years. The total number of patients with NF-pNET increased with time (*n* = 833 to *n* = 2644). Both sexes were almost equally represented (46.0% female and 54.0% male). Most patients were Caucasian (*n* = 3747, 78.6%), and the proportion remained steady during the study period (*n* = 679, 81.5% to *n* = 2023, 76.5%). Overall, most of the patients had distant disease (*n* = 2408, 50.5%) classified by the SEER historic stage, but the proportion decreased significantly during our study period (61.5 to 44.4%; *P* = 0.01). In addition, both the number and proportion of patients with localized disease displayed a rapid increase during the study period (*n* = 115, 13.8% to *n* = 991, 37.5%; *P* < 0.01). However, the number and proportion of patients with regional disease remained constant. Moreover, both the number and proportion patients with well-differentiated tumours markedly increased during our study period (*n* = 107, 12.9% to *n* = 1224, 46.3%; *P* < 0.01). Furthermore, the median tumour size (2004+) was 3.5 cm (*n* = 3535, 74.2%).

**Table 1:** Demographic and Pathological Characteristics of the Study Population (2000–2014)

Variable	Total	2000–2004	2005–2009	2010–2014
No. of patients	4766	833	1289	2644
Age of diagnosis	59	57	58	60
Gender				
Women	2192 (46.0)	391 (46.9)	606 (47.0)	1195 (45.2)
Men	2574 (54.0)	442 (53.1)	683 (53.0)	1449 (54.8)
Ethnicity				
White	3747 (78.6)	679 (81.5)	1045 (81.1)	2023 (76.5)
Black	583 (12.2)	95 (11.4)	150 (11.6)	338 (12.8)
Other†	408 (8.6)	56 (6.7)	89 (6.9)	263 (10.0)
Unknow	28 (0.6)	3 (0.4)	5 (0.4)	20 (0.8)
SEER historic stage				
Localized	1365 (28.6)	115 (13.8)	259 (20.1)	991 (37.5)
Regional	829 (17.4)	148 (17.8)	260 (20.2)	421 (15.9)
Distant	2408 (50.5)	512 (61.5)	723 (56.1)	1173 (44.4)
Unstage	164 (3.4)	58 (7.0)	47 (3.7)	59 (2.2)
Grade				
Well differentiated	1696 (35.6)	107 (12.9)	365 (28.3)	1224 (46.3)
Moderately differentiate	538 (11.3)	79 (9.5)	115 (8.9)	344 (13.0)
Poorly differentiated	309 (6.5)	71 (8.5)	86 (6.7)	152 (5.8)
Undifferentaited	88 (1.85)	27 (3.2)	14 (1.1)	47 (1.8)
Unknow	2135 (44.8)	549 (65.9)	709 (55.0)	877 (33.2)

†American Indian/AK Native, Asian/Pacific Islander

### Overall trends in incidence and IB mortality

**Fig. 2:**
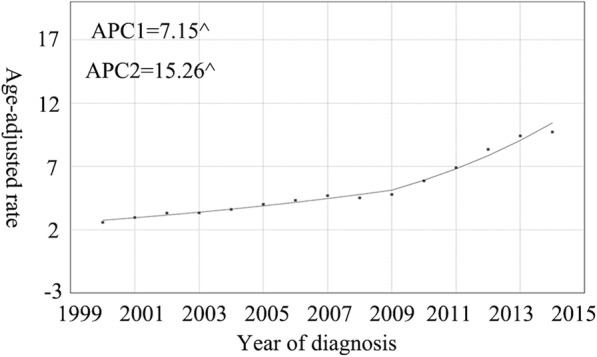
The incidence trends of NF-pNETs in SEER 18 overall 2000–2014. Graph shows from 2009 to 2014, the rate of increase in the incidence of this disease was particularly high

**Fig. 3:**
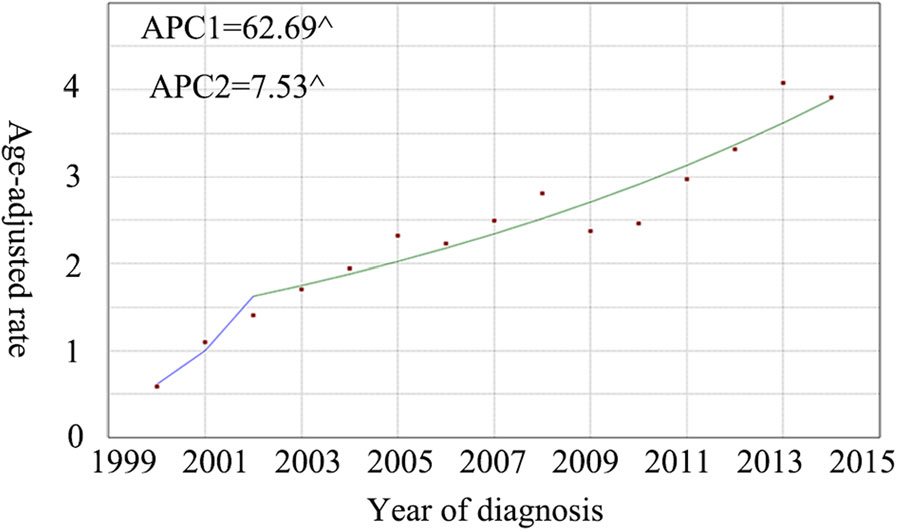
The IB mortality trends of SEER 18 overall 2000–2014. Graph shows that from 2002 to 2014, the APC of IB mortality decreased

Overall, the incidence of NF-pNETs increased (Fig. 2). In 2000, the incidence was 2.6 per 1,000,000 individuals, while in 2014, it increased to 9.7 per 1,000,000 individuals. From 2009 to 2014, the rate of increase in the incidence of this disease was particularly high. The APC (i.e., the extent of increase in incidence) for the incidence of NF-pNET from 2000 to 2009 was 7.2% (95% CI: 5.7–8.7; *P* < 0.01), whereas from 2010 to 2014, the APC was 15.3% (95% CI: 11.4–19.7; *P* < 0.01). Although the IB mortality of NF-pNET displayed a similar increase during the study period, from 0.6 per 1,000,000 individuals to 3.9 per 1,000,000 individuals, the trend was different (Fig. 3). From 2000 to 2002, the APC was 62.7% (95% CI: 19.6–121.4; *P* < 0.01), which was indeed alarming. However, from 2003 to 2014, the rate of change of IB mortality decreased, with an APC of 7.5% (95% CI: 5.6–9.5; *P* < 0.01).

### Trends by sex

As for the sex-specific incidence, the overall incidence in females was lower than that in males, but the overall incidence increased in both sexes. The incidence in males and females was 3.6 and 1.9 per 1,000,000 individuals in 2000 and 11.6 and 8.2 per 1,000,000 individuals in 2014, respectively. The APC was 6.5% (95% CI: 5.0–8.1; *P* < 0.01) in males from 2000 to 2009, whereas it was 5.5% (95% CI: 1.1–10.0; *P* < 0.01) in females from 2002 to 2009. Although there were some differences in the change of incidence in males and females, the rate of change of incidence increased in both sexes from 2010 to 2014, with a higher rate of increase in females than in males. From 2010 to 2014, the APC was 15.7% (95% CI: 11.6–19.9; *P* < 0.01) for males and 16.4% (95% CI: 10.1–23.1; *P* < 0.01) for females (Additional file 1: Figure S1a, b). In both sexes, the IB mortality increased during the study period. In contrast, the rate of change of IB mortality decreased in recent years in females, with an APC of 6.6% (95% CI: 4.7–8.5; *P* < 0.01) from 2002 to 2014 (Additional file 1: Figure S1 c,d).

### Trends by stage

The incidence of disease of all stages displayed an overall increase from 2000 to 2014. Distant disease accounted for a larger number of cases in 2000 than did localized disease (0.4 cases per 1,000,000 individuals for localized disease vs 1.4 cases per 1,000,000 individuals for distant disease). However, the trend was reversed in 2014 (4.2 cases per 1,000,000 individuals for localized disease vs 3.6 cases per 1,000,000 individuals for distant disease). The incidence of localized disease initially increased with an APC of 14.7% (95% CI: 10.4–19.2; *P* < 0.01) and displayed the largest rate of increase from 2009 to 2014, with an APC 31.9% (95% CI: 20.1–44.8; *P* < 0.01). However, the incidence of both regional and distant disease displayed a steady increase during the study period (Additional file 2: Figure S2a;b). Regarding IB mortality for NF-pNET, there was a steady increase in localized disease, with an APC of 19.8% (95% CI: 15.1–24.6; *P* < 0.01). In contrast, the rate of change in IB mortality for distant disease slowed down from 2003 to 2014, with an APC of 5.8% (95% CI: 3.8–7.9; *P* < 0.01), as did that for regional disease from 2002 to 2014, with an APC of 8.1% (95% CI: 3.0–13.4; *P* < 0.01) (Additional file 2: Figure S2c;d).

### Trends by tumour location

Tumour location is a significant factor for pancreatic lesions. Overall, the incidence of NF-pNETs located in the pancreatic head significantly increased, with an APC of 4.9% from 2000 to 2009 and 12.9% from 2010 to 2014 (Additional file 3: Figure S3). The incidence of tumours in other locations in the pancreas also displayed a steady increase. Regarding trends in IB mortality, there was an initial increase for patients with tumours in all locations of the pancreas, but IB mortality for patients with tumours in the tail of the pancreas decelerated after 2002 (Additional file 4: Figure S4).

### Trends by different pathological types

NF-pNET has many pathological subtypes. The incidence of Islet cell adenocarcinoma decreased in recent years, with an APC of − 7.7% (95% CI: (− 9.8)-(− 5.5); *P* < 0.01). However, the incidence of neuroendocrine carcinoma and carcinoid tumours displayed an APC of 8.9 and 22.3%, respectively (Table 2).

**Table 2:** Trends byDifferent Pathological Types

	Incidence APC	IB mortality APC
Large cell neuroendocrine	^a^	^a^
Islet cell adenocarcinoma	−7.7% (*P* < 0.01)	2.2% (*P* = 0.3)
Neuroendocrine carcinoma	8.9% (*P* < 0.01)	1.4% (*P* = 0.5)
carcinoid	22.3% (*P* < 0.01)	2.4% (*P* = 0.1)

Neuroendocrine in pancreas (malignant)

^a^can’t find enough statistics

### Trends by Cancer-directed surgical treatment

Among all 4766 included cases, 2436 (52.1%) underwent surgery (resection), and the proportion of patients with localized, regional, and distant disease undergoing surgery was 48.2, 27.4, and 23.2%, respectively. The proportion of patients undergoing surgery displayed a significant increase during the study period (37.2–55.1%; *P* < 0.01). Similarly, the proportion of patients with localized disease undergoing surgery substantially increased during the study period (31.0–56.3%, *P* < 0.01). In contrast, the proportion of patients with distant disease undergoing surgery significantly decreased during the study period (27.7–20.8%, *P* < 0.01). Overall, the proportion of patients undergoing surgery was lower for tumours in the head of the pancreas than for tumours in the tail of the pancreas (47.0% vs 60.7%; *P* = 0.047).

### Long-term survival outcomes

**Fig. 4:**
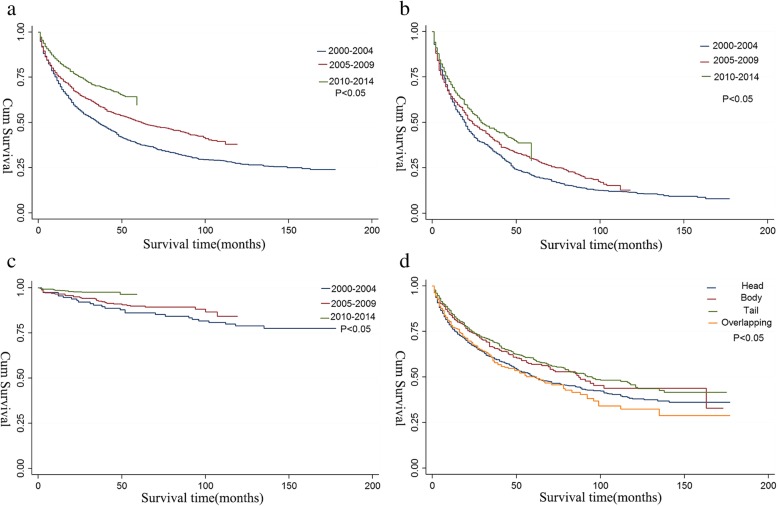
Kaplan-Meier’s analysis of NF-pNETs: **a** Survival time in study duration, Graph shows the median survival duration increased with time; **b** Survival time of distant disease; patients with distant disease displayed an increase in survival duration from 2000 to 2014; **c** Survival time of localized disease; patients with localized disease also displayed prolonged survival over time; **d** Survival time of different sites of pancreas; Graph shows that patients with tumours in the tail of the pancreas had the better median survival than patients with tumours in other sites. The *P* values reported for trend analysis refers to comparison among all of the sections. Abbreviation: Cum, cumulative

The median survival was 66 months (95% CI: 60–73), and the 1-, 5-, 10-year survival rates were 79.0, 51.8, and 38.1%, respectively. The median survival duration increased with time (Fig. 4a). Patients with distant disease displayed an increase in survival duration from 2000 to 2014 (20–29 months; *P* < 0.01; Fig. 4b). Patients with localized disease also displayed prolonged survival (Fig. 4c). Regarding tumour site, patients with tumours in the tail of the pancreas had the better median survival than patients with tumours in other sites (median survival, 95 months, 95% CI: 81–121 months; Fig. 4d).

Based on multivariable Cox regression analysis adjusted for patient clinical and demographic characteristics, tumour characteristics, treatment, age (> 59 years), tumour grade (moderately and poorly differentiated), stage at diagnosis (regional and distant), tumour size (> 2 cm), tumour site (body or tail of the pancreas) and resection were associated with mortality (Table 3).

**Table 3:** Multivariate Cox’s Proportional Hazards Model Assessing Factors Associated With Mortality After Diagnosis

Risk Factors	HR†	95%CI		*P* Vaule
		Lower	Upper	
Age,group by median				
0–59	Referent			
> 59	1.70	1.55	1.86	< 0.001
Gender				
Female	Referent			
Male	1.17	1.07	1.28	0.001
Ethnicity				
Other	Referent			
White	1.16	0.97	1.378	0.105
Black	1.21	0.98	1.50	0.072
Grade				
Well differentiated, I	Referent			
Moderately differentiated, II	2.09	1.71	2.57	< 0.001
Poorly differentiated;,III	8.31	6.91	9.98	< 0.001
Undifferentiated, IV	8.53	6.32	11.52	< 0.001
Stage				
Localized	Referent			
Regional	4.73	3.63	6.15	< 0.001
Distant	15.78	12.47	19.96	< 0.001
Tumor size, cm				
< =2 cm	Referent			
> 2	1.77	1.51	2.04	< 0.001
Location				
Head of pancreas	Referent			
Body of pancreas	0.80	0.68	0.94	0.007
Tail of pancreas	0.87	0.82	0.92	< 0.001
Overlapping lesion of pancreas	1.01	0.96	1.07	0.666
Treatment				
No surgery procedure	Referent			
Resection	0.15	0.13	0.16	< 0.001

†HRs greater than 1.0 indicates a higher risk of death

In particular, tumour in the body (hazard ratio [HR] = 0.80; 95% CI: 0.68–0.94; *P* < 0.007) and tail of the pancreas (HR = 0.87; 95% CI: 0.82–0.92; *P* < 0.001) vs tumour in the head of the pancreas and surgery vs no surgery (hazard ratio HR = 0.15; 95% CI: 0.13–0.16; *P* < 0.001) were associated with favourable prognoses.

## Discussion

NF-pNETs account for a certain proportion of pancreatic tumours [8]. Some pathological types of NF-pNETs are also fatal [14, 17, 18]. Therefore, the epidemiology, treatment and prognosis of the disease are worth studying. RR Salem [19] have previously studied the incidence and predictors of survival for small NF-pNETs (< 2 cm). Zhou H [20] explored the impact of ethnicity on this disease; AJ Moser [16] reported the epidemiological and prognostic factors of NF-pNET from 1973 to 2004; in addition, many studies have focused on the incidence of NF-pNETs but lack data on annual changes. It is important to improve our understanding of the epidemiology, natural history and prognostic factors associated with this disease.

To the best of our knowledge, this is the largest population-based study using the SEER database from 2000 to 2014 focused on the incidence and prognosis of NF-pNET in the US. An updated evaluation of the incidence and prognosis, as well as trends in increasing incidence and improved survival, was presented over time.

This study demonstrated that the incidence of NF-pNET is increasing in the US population. In recent years (from 2009 to 2014), the rate of increase in incidence has been significantly high. The acceleration of increase in the incidence of localized disease and well-differentiated tumours without changes in biological behaviours may be indicative of recent improvements in detecting pNETs [21–23] such as the more frequent use of axial imaging and endoscopic ultrasound and good understanding and classification [11] of NF-pNETs. However, although we demonstrated an acceleration of increase in the incidence of NF-pNETs from 2009 to 2014, the IB mortality presented an deceleration of increase trends in recent years. The deceleration of increase trends in IB mortality was also observed for distant disease. In addition, the median survival over time and all stages is increasing, indicated that a progressive increase in the detection of NF-pNET and appropriate therapy in recent years have led to a better response, resulting in good prognoses. Our finding of deceleration of increase trends IB mortality for NF-pNET is novel, contrary to the improved survival demonstrated in many studies [16, 19].

The higher rate of increase in the incidence of localized disease than distant disease is indicative of progressive improvements in the detection of NF-pNETs at an early stage and recent advancements in the treatment of distant disease. Additionally, the IB mortality for distant disease decelerate in increase trend. However, the rate of surgery in patients with distant disease significantly decreased during the study period, indicating that recent emergence of curative intervention with non-surgical treatments [3, 24], such as targeted therapy, chemotherapy, and radiotherapy, has improved the survival of patients with distant disease.

In this study, the increase in incidence was the highest in recent years, and African-American patients displayed the highest incidence. In contrast, both African-American and Caucasian patients displayed a deceleration in increase trend in IB mortality. The possible reasons may be the development of technologies that increased the detection of the disease so that it could be treated at an earlier stage; additionally, early treatment of the disease is more effective than later treatment.

The site of pNETs and NF-pNETs is a known factor that is essential for correct patient management [25], and it is also an important predictor of outcomes. Despite a steady increase in incidence, trend of the IB mortality for patients with tumours in the tail of the pancreas decelerated in recent years, and this tumour location presented the best outcome, indicating that treatment of tumours located in the tail of the pancreas is worth affirmation. On the other hand, tumours located in the head of the pancreas had the worst outcome, incidence and IB mortality, and tumours in other sites except tail did not present a favourable outcome either, indicating that the treatment of NF-pNETs in sites other than the tail have not displayed ideal therapeutic effects in recent years, and better treatment adjustment is needed to improve prognosis.

In our study, tumour size> 2 cm was independently associated with an increased risk of death. Yoji Kishi [26] found that for tumour size> 1.5 cm, the risk of metastasis and recurrence significantly increases, but for tumour size< 1.5 cm, there is little risk of metastasis and recurrence. Eric J [19] found that nodal metastasis occurs in a small proportion of small NF-pNETs (< 2 cm). Thus, consistent with other studies, small tumour size is associated with better survival [3, 17, 19, 26, 27].

Based on our results, patients undergoing surgery for primary and metastatic tumours had a prolonged survival. Although many factors have been included in our study, younger patients, patients with well- or moderately differentiated tumours and patients with favourable tumour location were more inclined to undergo surgery. It has also been confirmed that surgery can benefit to patients with distant disease [25].

Nevertheless, our study has several limitations. The SEER database does not provide information on patient comorbidities and clinical details such as biliary obstruction, which may affect prognosis. In any case, we focused on all-cause mortality, and our results may not have been influenced by this limitation. There are many differences between mortality and survival; however, bias in survival analysis does not affect mortality analysis [28]. In addition, patient selection bias, miscoding, and incorrect data classification should be considered. However, it has been verified that misclassification is very minimal in the SEER database [29–31]. Furthermore, we did not include detailed information on adjuvant treatment such as chemotherapy. Finally, data on tumour size was only obtained after 2004.

## Conclusions

The diagnosis and treatment of NF-pNET are changing in the US, with favourable results based on stage, treatment, survival and mortality. Although the incidence of this disease has increased in recent years, IB mortality has a deceleration in increase trend, indicating recent improvements in detecting NF-pNETs on one hand and better treatment of this disease on the other. Even for cases with distant disease, non-surgical treatments have improved prognosis. Moreover, in contrast to that for tumours in the pancreatic head, the treatment for tumours the pancreatic tail has improved, suggesting that there is room for further improvement of survival for patients with tumours in the head of the pancreas. Therefore, early diagnosis and appropriate treatment adjustments continue to be a priority in improving patient outcomes.

## Additional files

Additional file 1: **Figure S1.** APC of incidence trend and IB mortality trend in gender: a and b. APC incidence trend of in men (a) and women (b). APC of incidence trend increased obviously after 2009 in both man and women; c and d. APC of IB mortality trend in men(c) and women (d). APC change of IB mortality decreased in recent years in females. (TIF 382 kb) (TIF 387 kb)

Additional file 2: **Figure S2.** APC of incidence trend and IB mortality trend in disease stages: a. APC of incidence trend in localized disease. The incidence of localized disease displayed the largest rate of increase from 2009 to 2014; b. APC of incidence trend in regional and distant disease. The incidence of both regional and distant disease displayed a steady increase during the study period; c. APC of IB mortality trend of different stage of localized disease. There was a steady increase in localized disease; d. APC of IB mortality trend of regional and distant disease. The rate of change in IB mortality for distant disease slowed down from 2003 to 2014, as did that for regional disease from 2002 to 2014. (TIF 409 kb)

Additional file 3: **Figure S3.** APC of incidence trend in anatomical region of pancreas: a. APC of incidence trend in disease located body of pancreas. The incidence of tumours in body of pancreas displayed a steady increase; b. APC of incidence trend in disease located head of pancreas. The incidence of NF-pNETs located in the pancreatic head significantly increased; c. APC of incidence trend in disease located tail of pancreas. The incidence of tumours in tail of pancreas keeps steady increase with APC 13.78%; d. APC of incidence trend in disease located overlapping site of pancreas. The incidence of tumours in overlapping of pancreas keeps steady increase. (TIF 382 kb)

Additional file 4: **Figure S4.** APC of IB mortality trend in anatomical region of pancreas: a. APC of IB mortality trend in disease located body of pancreas; b. APC of IB mortality trend in disease located head of pancreas; c. APC of IB mortality trend in disease located tail of pancreas; d. APC of IB mortality trend in disease located overlapping site of pancreas. There was an initial increase for patients with tumours in all locations of the pancreas, but IB mortality for patients with tumours in the tail of the pancreas decelerated after 2002. (TIF 386 kb)

## Abbreviations

APC: Annual percentage changes
IB mortality: Incidence-based (IB) mortality
NF-pNETs: Non-functional pancreatic neuroendocrine tumours
SEER: Surveillance, Epidemiology, and End Results
