# Supplementary figures and images for: Non-functional pancreatic neuroendocrine tumours: emerging trends in incidence and mortality
Source: BMC Cancer. 2019 Apr 8;19:334. doi: 10.1186/s12885-019-5543-2 (PMC6454719; doi:10.1186/s12885-019-5543-2)

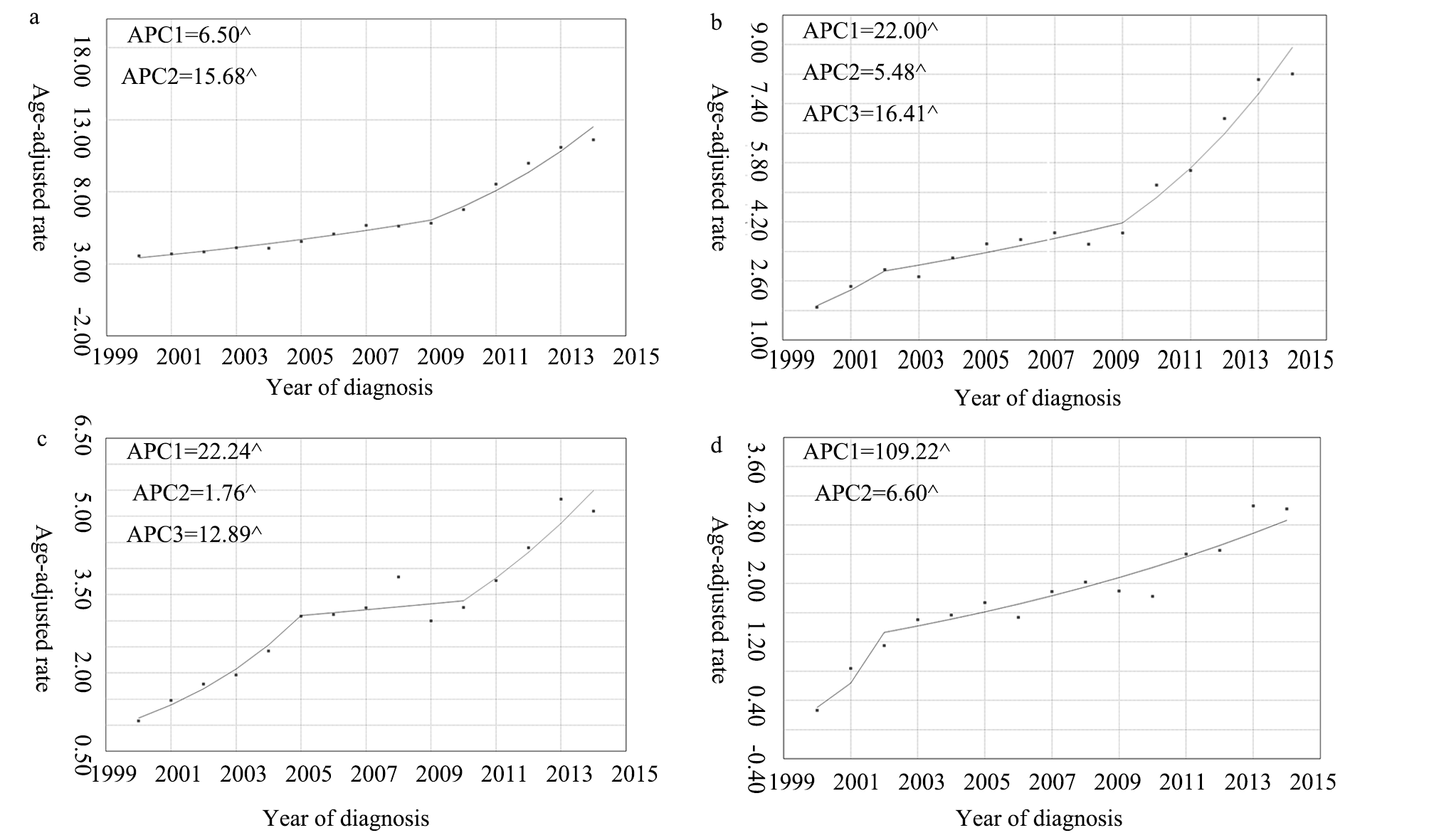

Supplement: Supplementary file 1 — Figure S1. APC of incidence trend and IB mortality trend in gender: a and b. APC incidence trend of in men (a) and women (b). APC of incidence trend increased obviously after 2009 in both man and women; c and d. APC of IB mortality trend in men(c) and women (d). APC change of IB mortality decreased in recent years in females. (TIF 382 kb) (TIF 387 kb) [file 12885_2019_5543_MOESM1_ESM.tif]

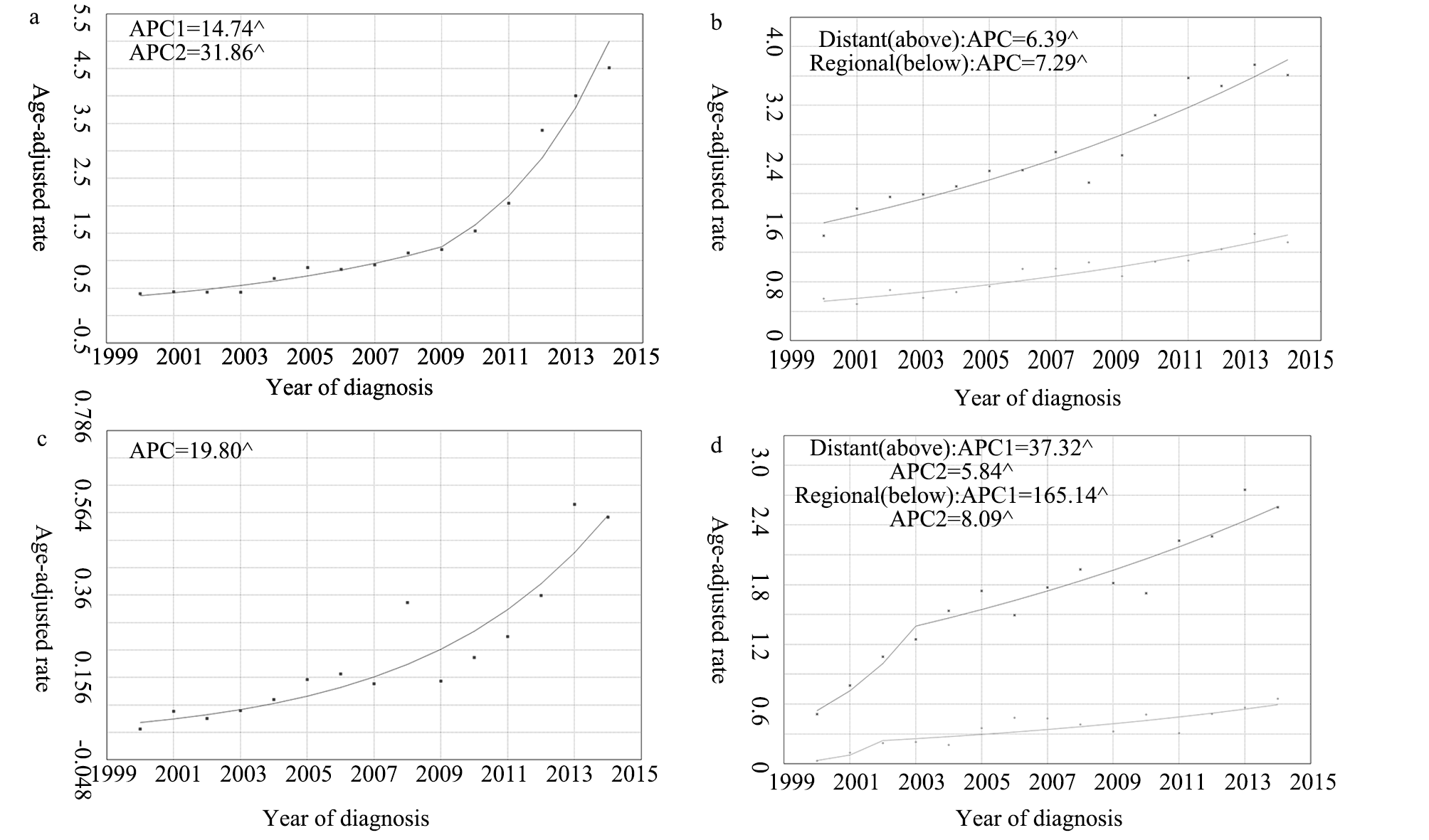

Supplement: Supplementary file 2 — Figure S2. APC of incidence trend and IB mortality trend in disease stages: a. APC of incidence trend in localized disease. The incidence of localized disease displayed the largest rate of increase from 2009 to 2014; b. APC of incidence trend in regional and distant disease. The incidence of both regional and distant disease displayed a steady increase during the study period; c. APC of IB mortality trend of different stage of localized disease. There was a steady increase in localized disease; d. APC of IB mortality trend of regional and distant disease. The rate of change in IB mortality for distant disease slowed down from 2003 to 2014, as did that for regional disease from 2002 to 2014. (TIF 409 kb) [file 12885_2019_5543_MOESM2_ESM.tif]

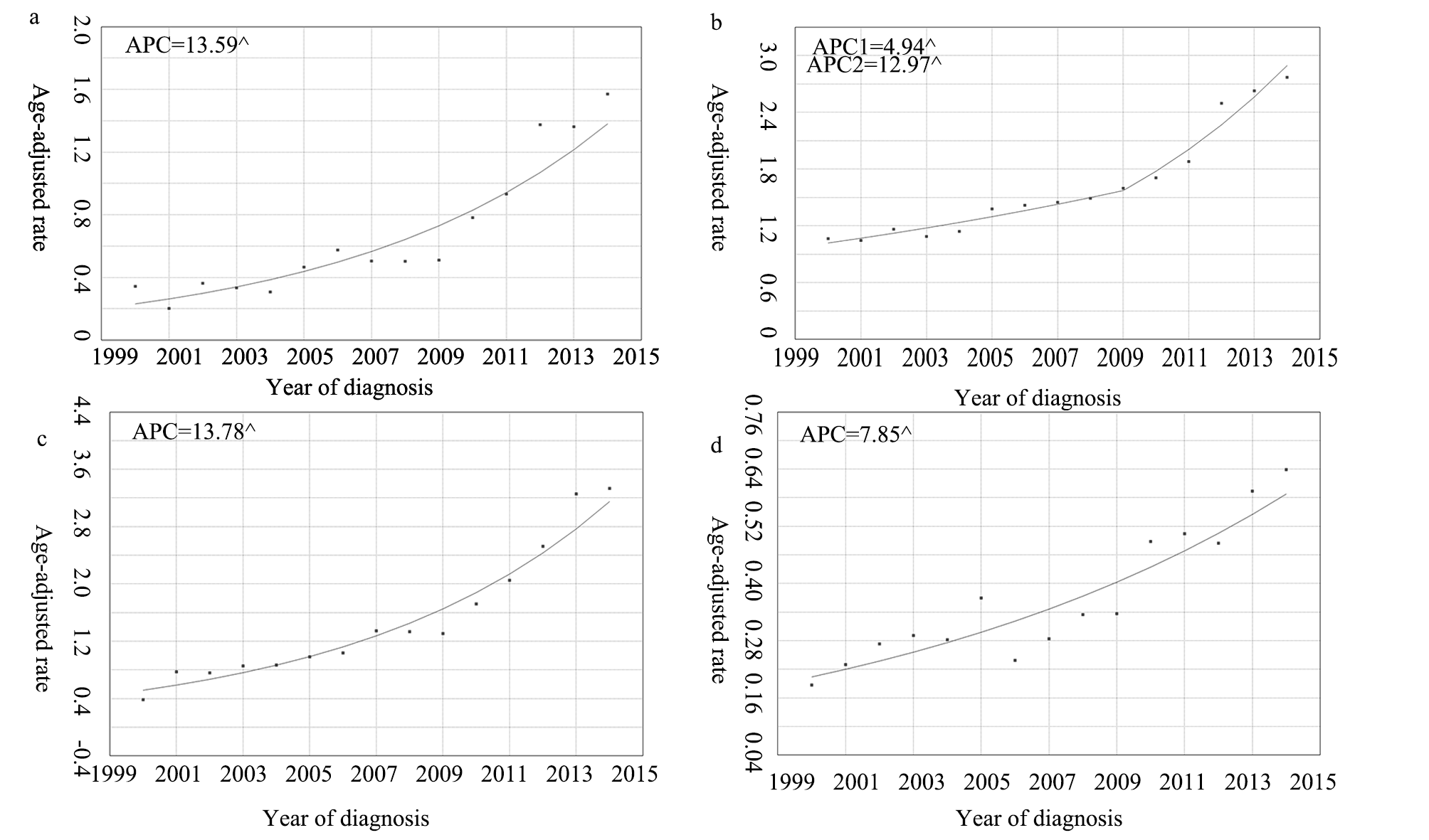

Supplement: Supplementary file 3 — Figure S3. APC of incidence trend in anatomical region of pancreas: a. APC of incidence trend in disease located body of pancreas. The incidence of tumours in body of pancreas displayed a steady increase; b. APC of incidence trend in disease located head of pancreas. The incidence of NF-pNETs located in the pancreatic head significantly increased; c. APC of incidence trend in disease located tail of pancreas. The incidence of tumours in tail of pancreas keeps steady increase with APC 13.78%; d. APC of incidence trend in disease located overlapping site of pancreas. The incidence of tumours in overlapping of pancreas keeps steady increase. (TIF 382 kb) [file 12885_2019_5543_MOESM3_ESM.tif]

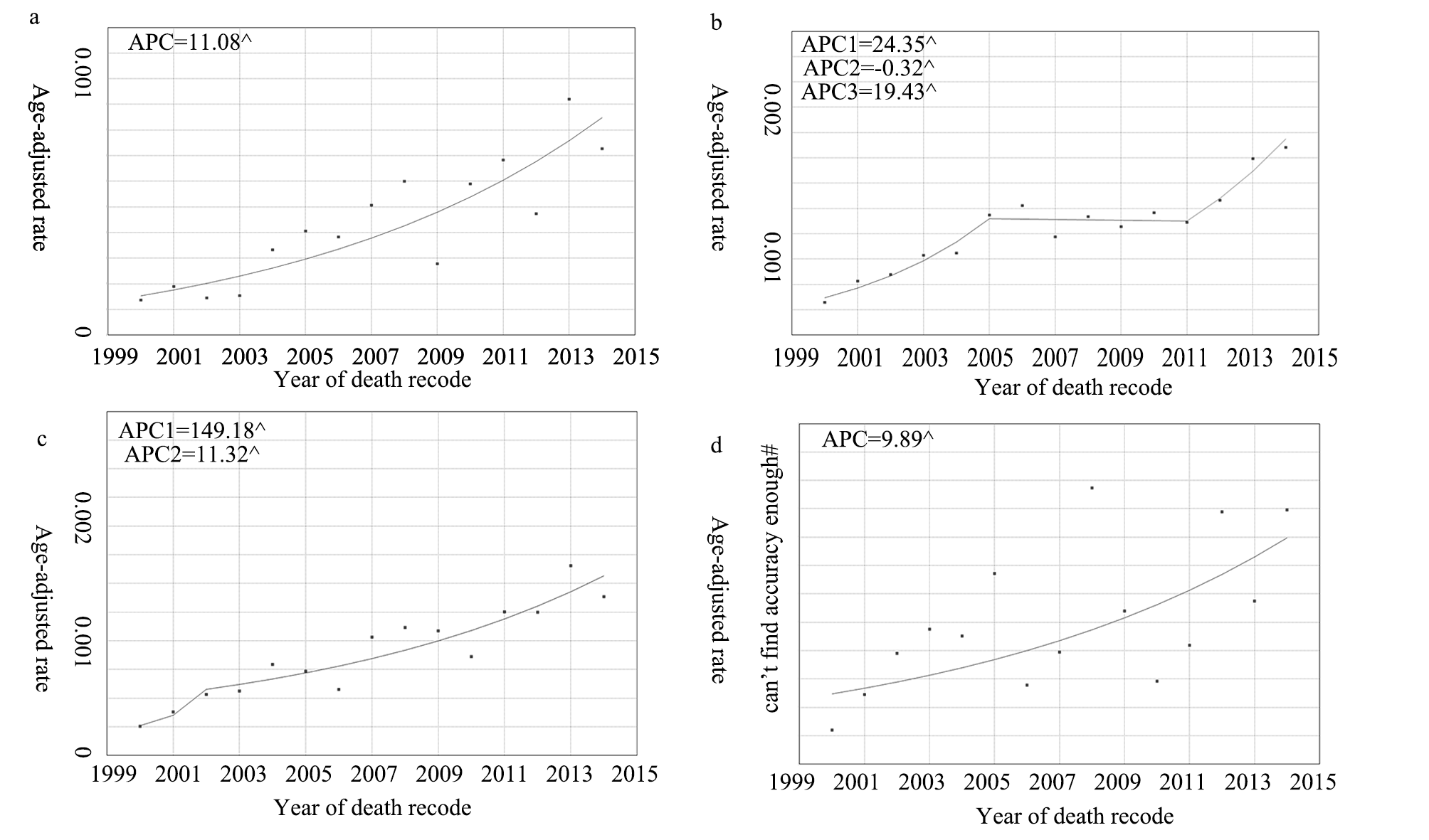

Supplement: Supplementary file 4 — Figure S4. APC of IB mortality trend in anatomical region of pancreas: a. APC of IB mortality trend in disease located body of pancreas; b. APC of IB mortality trend in disease located head of pancreas; c. APC of IB mortality trend in disease located tail of pancreas; d. APC of IB mortality trend in disease located overlapping site of pancreas. There was an initial increase for patients with tumours in all locations of the pancreas, but IB mortality for patients with tumours in the tail of the pancreas decelerated after 2002. (TIF 386 kb) [file 12885_2019_5543_MOESM4_ESM.tif]
